# Supplementary material for: Impedimetric Polyaniline-Based Aptasensor for Aflatoxin B1 Determination in Agricultural Products
Source: Foods. 2023 Apr 19;12(8):1698. doi: 10.3390/foods12081698 (PMC10137590; doi:10.3390/foods12081698)
Supplement: Supplementary file 1 [file foods-12-01698-s001.zip › foods-2350646-supplementary.pdf]

Supplementary Material

Impedimetric Polyaniline-based Aptasensor for Aflatoxin B_1 Determination in Agricultural Products

Jing Yi Ong [1], Sook-Wai Phang [2], Choo Ta Goh [1], Andrew Pike [3] and Ling Ling Tan [1,*]

[1] Southeast Asia Disaster Prevention Research Initiative (SEADPRI), Institute for Environment and Development (LESTARI), Universiti Kebangsaan Malaysia, 43600 UKM Bangi, Selangor Darul Ehsan, Malaysia

[2] Department of Physical Science, Faculty of Applied Sciences, Tunku Abdul Rahman University College, Jalan Genting Kelang, Setapak, 53300 Kuala Lumpur, Malaysia

[3] School of Natural and Environmental Sciences, Bedson Building, Newcastle University, Newcastle Upon Tyne NE1 7RU, UK

*Correspondence: lingling@ukm.edu.my; Tel.: +6012 2321300

Table S1

Electrochemical data of the cyclic voltammograms of PAni/Apt SPE at different scan rates between 50 mV s^{-1} and 300 mV s^{-1}.

Scan Rate (mV s^{-1})	$v^{1/2}$ (V s^{-1})$^{1/2}$	i_{pa} (×10^{-5} A)	i_{pc} (×10^{-5} A)	i_{pa}/i_{pc}	E_{pa} (V)	E_{pc} (V)	$E_{1/2}$ (V)	ΔE_p (V)
50	0.22	64.331	−38.113	1.688	0.452	−0.300	0.076	0.752
100	0.32	112.457	−97.504	1.153	0.670	−0.546	0.062	1.216
150	0.39	147.491	−149.139	0.989	0.783	−0.768	0.008	1.551
200	0.45	194.916	−197.266	0.988	0.835	−0.839	0.002	1.674
250	0.50	233.307	−233.429	0.999	0.911	−0.905	0.003	1.816
300	0.55	255.310	−255.219	1.000	0.954	−0.943	0.006	1.897

Table S2

Electrochemical data of anodic and cathodic current against the square root of scan rates for PAni-modified SPE.

$v^{1/2}$ [(V s^{-1})$^{1/2}$]	i_{pa} (µA)	i_{pc} (µA)
0.22	6.433	-3.811
0.32	11.246	-9.750
0.39	14.749	-14.914
0.45	19.492	-19.727
0.5	23.331	-23.343
0.55	25.531	-25.522

Table S3

Electrochemical data of anodic and cathodic potential against the logarithm of scan rate for PAni-modified SPE.

v (V s^{-1})	log v (V s^{-1})	E_{pa} (V)	E_{pc} (V)
0.05	-1.301	0.452	-0.300
0.10	-1.000	0.670	-0.546
0.15	-0.824	0.783	-0.768
0.20	-0.699	0.835	-0.839
0.25	-0.602	0.911	-0.905
0.30	-0.523	0.954	-0.943

Table S4

Electrochemical data of the cyclic voltammograms of bare SPE, PAni-modified SPE, PAni/Apt-modified SPE, and PAni/Apt-modified SPE during 5 nM AFB_1 detection at a scan rate of 100 mV s^{-1}.

Type of SPE	i_{pa} (×10^{-5} A)	i_{pc} (×10^{-5} A)	i_{pa}/i_{pc}	E_{pa} (V)	E_{pc} (V)	$E_{1/2}$ (V)	ΔE_p (V)
Blank SPE	4.602	-4.623	0.995	0.551	-0.111	0.220	0.662
PAni-Coated SPE	73.029	-73.578	0.993	0.632	-0.210	0.211	0.842
PAni/Apt-Coated SPE	58.496	-81.055	0.722	0.916	-0.797	0.060	1.713
5 nM AFB_1	48.871	-66.650	0.733	0.883	-0.782	0.051	1.665

Table S5

Electrochemical data obtained from the Nyquist plots of bare SPE, PAni-modified SPE, PAni/Apt-modified SPE and PAni/Apt-modified SPE during 5 nM AFB_1 detection in 13 mL of 5 mM $K_3[Fe(CN)_6]$ redox indicator containing 0.1 M KCl at room temperature (25 °C).

Type of SPE	R_{CT} (Ω)
Blank SPE	325.939
PAni-modified SPE	186.338
PAni/Apt-modified SPE	213.756
5 nM AFB_1 after detection	234.099

Table S6

Electrochemical data of the Nyquist plots of SPE coated with 3 µL and 4 µL of PAni in 5 mM $K_3[Fe(CN)_6]$ redox indicator containing 0.1 M KCl.

Type of SPE	R_{CT} (Ω)
Blank SPE	355.103
PAni-modified SPE (3uL)	226.713
PAni-modified SPE (4uL)	202.553

Table S7

Electrochemical data acquired from the Nyquist plots of the calibration curve for PAni/Apt SPE during the detection of AFB_1 with concentration ranging from 0.03 nM to 0.25 nM AFB_1 at pH 7.5.

AFB_1 Concentration (nM)	R_{CT} (Ω)			Average R_{CT} (Ω)	Standard Deviation	Relative Standard Deviation (%)
0.03	215.325	222.135	201.650	213.037	10.432	4.90
0.04	223.107	226.065	211.752	220.308	7.556	3.43
0.06	234.915	241.155	225.075	233.715	8.107	3.47
0.07	245.194	238.604	242.243	242.014	3.301	1.36
0.08	241.396	261.714	248.865	250.658	10.277	4.10
0.10	240.129	231.129	219.673	230.310	10.253	4.45
0.12	201.645	212.385	202.280	205.437	6.026	2.93
0.15	201.004	203.027	212.670	205.567	6.234	3.03
0.20	205.500	209.428	207.702	207.543	1.969	0.95
0.25	206.823	205.404	206.817	206.348	0.818	0.40

Table S8

Electrochemical data obtained from the Nyquist plots of PAni-based aptasensor during 0.05 nM AFB_1 detection (30 min) and the regeneration of aptasensor by incubating the aptasensor in 50 µM non-modified AFB_1 Apt solution as the regeneration solution for 30 min (*n*=4).

**Type of SPE**	**Detection 1**	**Detection 2**	**Detection 3**	**Detection 4**	**Average R_{CT} (Ω)**	**Standard Deviation**	**Repeatability relative standard deviation (%)**
**Aptasensor 1**	237.084	244.417	250.045	260.396	247.986	9.829	3.964
**Aptasensor 2**	236.650	239.322	244.027	256.821	244.205	8.946	3.663
**Aptasensor 3**	230.239	242.902	245.787	255.795	243.681	10.527	4.320
**Aptasensor 4**	228.079	232.320	239.034	250.528	237.490	9.792	4.123
**Average R_{CT} (Ω)**	233.013	239.740	244.723	255.885	-	-	-
**Standard Deviation**	4.540	5.388	4.557	4.080	-	-	-
**Reproducibility relative standard deviation (%)**	1.948	2.248	1.862	1.594	-	-	-

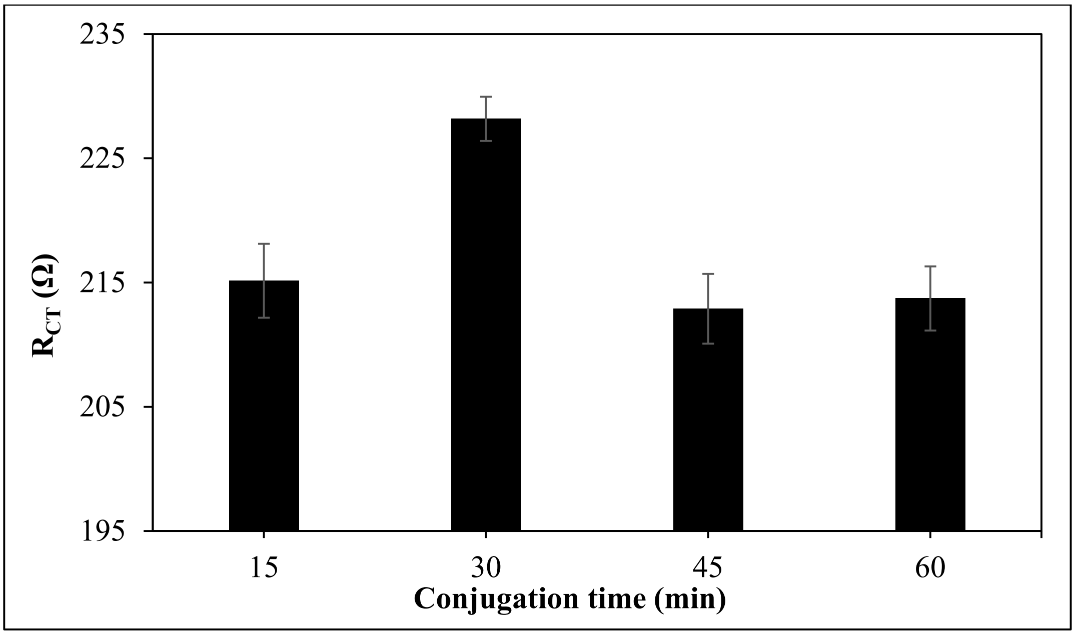

Figure S1. Bar graph of the AFB_1 aptasensor during 0.05 nM AFB_1 detection at different conjugation times of 15, 30, 45, and 60 min in 5 mM $K_3[Fe(CN)_6]$ redox indicator containing 0.1 M KCl at room temperature. The error bars indicate ± standard deviation, *n*=3.

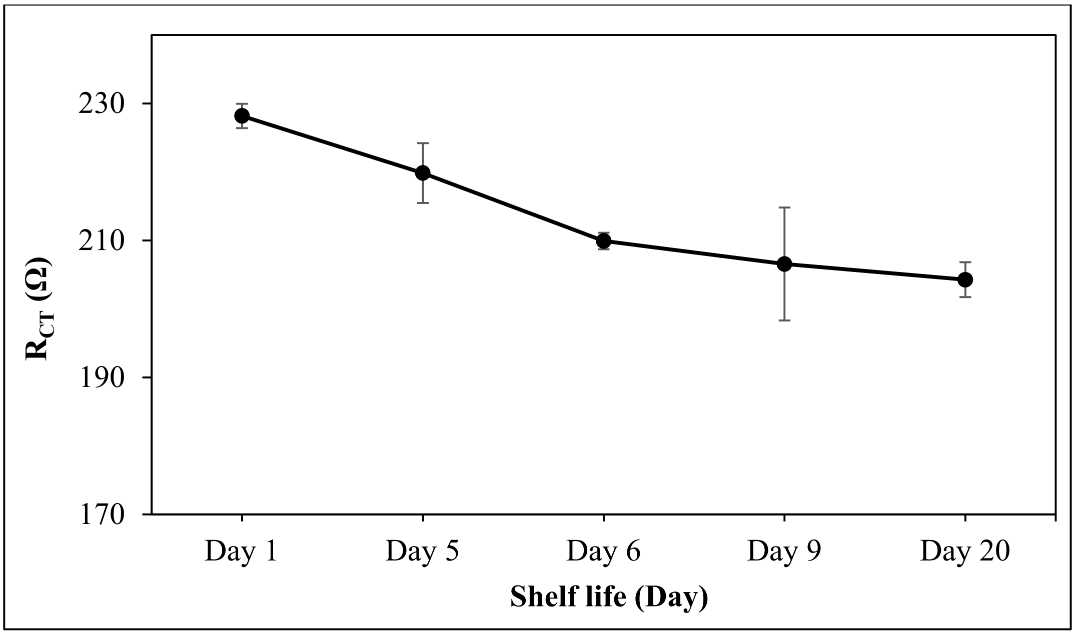

Figure S2. The line graph of PAni/Apt SPEs that are stored at 4 °C during the detection of 0.05 nM AFB_1 over 20 days in 5 mM $K_3[Fe(CN)_6]$ redox indicator containing 0.1 M KCl at pH 7.5 to determine the level of long-term stability of the aptasensor. The error bars indicate standard deviation, *n*=3.
